# All Eyes and Ears: A Case of Neurosyphilis Presenting With Simultaneous Ocular and Otic Involvement

**DOI:** 10.7759/cureus.56492

**Published:** 2024-03-19

**Authors:** Derek Zhang, Asma Safa Bahrami, Wilson D Ricketts

**Affiliations:** 1 Internal Medicine-Pediatrics, University of California Los Angeles, Los Angeles, USA

**Keywords:** tinnitus, penicillin g, immunocompetent, sexually transmitted infections, ocular syphilis, otosyphilis, secondary syphilis, syphilis

## Abstract

This report details a case of neurosyphilis manifesting as concurrent ocular and otosyphilis, an uncommon presentation of the disease. Here, we describe the diagnosis and treatment of a 27-year-old immunocompetent Caucasian male who presented with uveitis and tinnitus.

Physical exam was consistent with uveitis and audiometric testing revealed bilateral sensorineural hearing loss. Serum rapid plasma reagin (RPR) was reactive at 1:512 with a follow-up cerebrospinal fluid (CSF) venereal disease research laboratory (VDRL) test likewise reactive at 1:2, confirming neurosyphilis. The patient was treated with intravenous penicillin G with improvement of symptoms and with subsequent improvement of serum and CSF RPR. However, he ultimately represented with recurrent symptoms and fluctuating serum RPR levels, necessitating repeat treatment and ongoing clinical monitoring.

Neurosyphilis can occur at any point during the course of a syphilis infection and may present with a variety of nonspecific findings. This case documents a particularly uncommon instance of simultaneous ocular and otosyphilis, a presentation of neurosyphilis that has only been described a handful of times.

## Introduction

Syphilis is a sexually transmitted infection caused by the spirochete *Treponema pallidum*. Known colloquially as "the great imitator," syphilitic infections present with a wide variety of clinical manifestations ranging from localized, painless chancres to multi-organ disease. Since its historic low in 2001, reports of syphilis infection in the United States have been progressively increasing. Accurate epidemiologic data for syphilis is difficult to obtain due to under-detection, but, using reported cases from 2008 along with mathematical modeling, researchers from the Centers for Disease Control and Prevention (CDC) estimated the number of prevalent and incident syphilis infections in the United States in 2018 to be 156,000 and 146,000, respectively [1]. This represents an approximate 300% increase in syphilis cases from 2008, with the majority of cases occurring in the men who have sex with men (MSM) population, a reminder of the significant health disparities faced by lesbian, gay, bisexual, transgender, and queer (LGBTQ) patients in particular [1]. Due to its wide range of presentations, increasing prevalence, and the risk of long-term sequelae stemming from untreated infection, maintaining a high diagnostic suspicion for syphilis as well as a familiarity with its protean character is crucial for clinicians.

Neurosyphilis, a treponemal infection involving the central nervous system (CNS), is one of the most potentially devastating of the disease's many forms. Neurosyphilis can occur at any point during a syphilis infection and is characterized by a wide variety of symptoms ranging from mild meningismus in early stages, to paresis, stroke-like syndromes, or dementia in chronic untreated cases [2,3]. Ocular and otosyphilis are subtypes of neurosyphilis that present with primarily ophthalmologic and audiovestibular findings, respectively. The most common presentation of ocular syphilis is panuveitis, with postinfectious complications including cataracts, chorioretinitis, and posterior synechiae [4]. Otosyphilis typically presents with hearing loss, tinnitus, and vertigo [5]. Both ocular and otologic forms of neurosyphilis are rare manifestations of syphilis, together encompassing less than 2% of reported cases [6]. Here, we present a particularly unusual case of concurrent ocular and otologic neurosyphilis in an immunocompetent young adult.

## Case presentation

A 27-year-old Caucasian male with no significant past medical history presented to the outpatient clinic for evaluation of facial flushing which had been present for three months.

He reported that he had also been experiencing several other symptoms that had begun around the same time as the facial flushing: an urticarial rash that started on his back and extended outwards over his trunk, limbs, and face, as well as the subsequent development of scleral injection, tinnitus, lightheadedness, and chronic intermittent pharyngitis. When the rash did not go away after three weeks, he went to an urgent care and was prescribed a five-day course of prednisolone, after which it resolved. However, two weeks later he subsequently developed bilateral scleral injection. He went to an out-of-state ophthalmologist who gave him steroid eye drops with initial improvement, but the scleral injection returned as soon as he stopped using the steroid drops. He then returned to a second ophthalmologist who diagnosed him with blepharitis and gave him a course of topical erythromycin which he was still taking at the time of presentation. In addition, he reported several months of worsening tinnitus.

Family history was noncontributory, but his social history was notable on two accounts: significant travel related to work, which had made it difficult for him to obtain continuous medical care, as well as the fact that he attended weekly electronic dance music shows and rock concerts without using hearing protection. 

His vitals were stable and unremarkable. His exam was notable for mildly plethoric facies, bilateral scleral injection with circumferential sparing around the iris (right greater than left), and mild pharyngeal erythema. Inguinal exam revealed multiple three-centimeter lymph nodes bilaterally that were nontender and nonfluctuant. The remainder of the genitourinary exam was otherwise unremarkable with no visible urethral discharge or penile lesions. The neurologic exam was also unremarkable.

**Figure 1:**
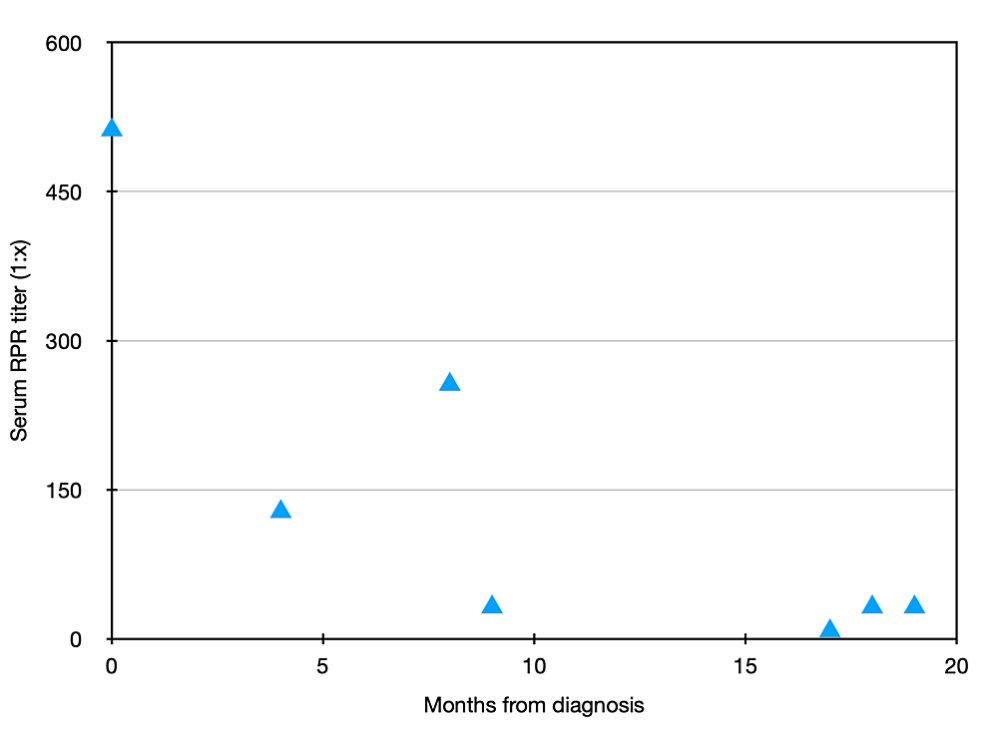
Serum RPR Trend Over Time RPR: Rapid plasma reagin

On further questioning, the patient revealed that he had had five female sexual partners in the last six months with intermittent condom use. Laboratory workup revealed a serum rapid plasma reagin (RPR) that was reactive at 1:512 (Figure 1). Human immunodeficiency virus (HIV), gonorrhea, and chlamydia tests were negative. Serum chemistries and complete blood counts were unremarkable as well. After discussing the case with the infectious disease attending on-call, the patient was advised to present to the emergency department for lumbar puncture and likely admission given that his symptoms of uveitis and tinnitus were highly concerning for neurosyphilis.

Although initially hesitant to return for further evaluation due to a busy work schedule, he ultimately presented to the emergency department the next week. Lumbar puncture was performed with cerebrospinal fluid (CSF) indices notable for elevated lymphocytes (92%) and elevated protein at 75 mg/dL (Table 1). The CSF venereal disease research laboratory (VDRL) test was reactive with a titer of 1:2, confirming neurosyphilis. The patient was admitted for placement of a peripherally inserted central catheter for administration of continuous intravenous penicillin G for a 14-day course, after which the patient experienced resolution of his ocular symptoms as well as his inguinal lymphadenopathy. His tinnitus also resolved but on audiology evaluation, he was found to have mild bilateral sensorineural hearing loss.

**Table 1:** CSF Findings CSF: Cerebrospinal fluid; RBC: red blood cell; TNC: total nucleated cell; VDRL: venereal disease research laboratory test; IgG: immunoglobulin G; IFA: immunofixation

Date	Segmented Neutrophils	Lymphocytes	Monocytes	Total Cells	CSF Appearance	RBC Count	TNC Count	Protein	Glucose	VDRL
03/15/22	1	92	7	100	Clear and colorless	65/cmm	20/cmm	75 mg/dL	54 mg/dL	Reactive
11/16/22	0	98	2	100	Clear and colorless	<1/cmm	3/cmm	55mg/dL (15-45 WNL)	61 mg/dL (43-73 WNL)	Nonreactive at 1:1, 1:2, and 1:4
08/25/23	0	94 (reactive lymphocytes seen)	6	100	Clear and colorless	1/cmm	2/cmm	53 mg/dL	62 mg/dL	Nonreactive at 1:1, 1:2, and 1:4 Treponema palladium Ab, IgG by IFA (CSF) Nonreactive

A follow-up serum RPR titer collected at four months from treatment down-trended to 1:128, but at six months the RPR up-trended again to 1:256. A repeat lumbar puncture was performed at this time with normal CSF indices and a nonreactive CSF VDRL. Repeat serum RPR one month later demonstrated a further decrease to 1:32, after which the patient was lost to follow-up. He then represented eight months later after he began experiencing increased fatigue, brain fog, decreased concentration, decreased exercise tolerance, and recurrent tinnitus. An initial serum RPR was repeated and found to be 1:8 but symptoms persisted and a repeat serum RPR two weeks after later increased to 1:32, prompting a repeat lumbar puncture which demonstrated CSF negative for VDRL but with elevated lymphocytes and protein. He was given one dose of IM PCN and treated with a course of oral doxycycline. Magnetic resonance imaging and magnetic resonance angiography brain and neck were obtained but were unremarkable. He also underwent a full ophthalmologic exam which was normal, as well as an expanded infectious workup at the recommendation of an infectious disease (ID) specialist. Serologic testing was negative for Borrelia burgdorferi, Rickettsiae species, Coccidioides species, Cryptococcus species, HIV 1/2, and West Nile virus. Repeat gonorrhea and chlamydia testing was also negative. Serum RPR remained elevated at 1:32. After discussion with the ID specialist, a plan was made to continue monitoring serum RPR at three-month intervals and, if up-trending again, to consider further treatment with another agent such as IV ceftriaxone.

## Discussion

Syphilitic infections generally follow a similar overarching course. The initial presentation of syphilis (primary syphilis) manifests as a solitary, painless chancre that typically localizes over the genitals [2]. Without treatment, primary syphilis progresses to secondary syphilis, correlating with hematogenous spread of the infection. Secondary syphilis is characterized by systemic symptoms that may include a highly variable rash that often mimics the rashes of other conditions, systemic symptoms such as fever and myalgias, or organ-specific involvement such as lymphadenopathy, hepatitis, or nephrotic syndrome [2]. Left untreated, syphilis progresses to tertiary syphilis, marked by the presence of cardiovascular disease (aortitis, coronary artery disease, aortic valve disease), the formation of gummas, or severe neurologic complications such as generalized paresis and tabes dorsalis. The complications of tertiary syphilis have been rare since the advent of penicillin [2]. 

Neurosyphilis specifically refers to *Treponema pallidum* infiltration into the CNS. It can occur at any point during the course of a syphilis infection. Early neurosyphilis can be asymptomatic or may present with meningeal symptoms such as headache, photophobia, and neck stiffness. Later manifestations of neurosyphilis may result in meningovascular disease resulting in strokes or stroke-like syndromes. Other late-stage complications may include parenchymal or spinal cord involvement, leading to generalized paresis, personality changes, or tabes dorsalis [2,3]. 

Neurosyphilis may alternatively present with more isolated sensory organ involvement, such as in ocular or otologic subtypes of neurosyphilis. The most common manifestation of ocular syphilis is panuveitis, though ocular syphilis can alternatively present with separate anterior or posterior uveitis, or optic neuritis [4]. Otologic syphilis, or otosyphilis, most commonly presents with rapid-onset sensorineural hearing loss, or with tinnitus and vertigo [5]. For some patients, hearing loss is permanent even after the infection is treated with antibiotics [5]. Neurosyphilis and its subtypes of ocular syphilis and otosyphilis are rare among patients with syphilis, though true prevalence may be underestimated due to under-reporting. A 2019 analysis of syphilis cases from 16 states found that ocular syphilis made up around 1% of cases, while otosyphilis made up 0.4% of cases [6]. 

Per the CDC guidelines, in patients with syphilis who demonstrate neurologic findings, CSF analysis should be obtained prior to initiating treatment [5]. CSF findings in neurosyphilis include pleocytosis, mildly elevated protein, and positive CSF VDRL [2,3]. Guideline-recommended treatment for neurosyphilis is the same regardless of clinical symptoms, with treatment being 10-14 days of intravenous penicillin G, administered either continuously or in increments of 3-4 million units every four hours [7]. To assess for disease resolution, the CDC recommends obtaining a follow-up serum RPR rather than repeat CSF analysis, as data suggest that RPR normalization predicts normalization of CSF parameters [7,8]. 

In our patient, the presentation of diffuse lymphadenopathy, pharyngitis, and urticarial rash is consistent with secondary syphilis. However, our patient’s scleral injection and tinnitus also suggest concurrent ocular and otologic syphilis. Instances of concurrent otologic and ocular syphilis, such as in our case, are exceedingly rare, with few case reports in the literature [9-11]. Our patient notably did not report any symptoms of early classical neurosyphilis, such as headache or photophobia, nor did his neurologic exam show any abnormalities. Instead, it was the patient’s positive serum RPR that recontextualized the patient’s presentation of tinnitus and scleral injection which ultimately prompted the workup for neurosyphilis.

## Conclusions

This case demonstrates the importance of considering less common presentations of syphilis when encountering patients with nonspecific symptoms but with pertinent risk factors, as well as underscoring the importance of routine screening for sexually transmitted infections. Although our patient presented with several symptoms that, when put in context, fit readily within an illness script for secondary syphilis, it could still have been possible to miss the true underlying diagnosis of neurosyphilis. For instance, it could have been all too easy to misattribute the patient's tinnitus to his habit of regularly attending multiple concerts without adequate hearing protection rather than recognizing that this actually represented the extension of the infection into the CNS. Without considering the less common forms of neurosyphilis such as ocular and otosyphilis, this patient might never have undergone a lumbar puncture, possibly resulting in delayed diagnosis and treatment, placing him at significant risk for permanent sensory damage. Thus, although ocular and otic manifestations of neurosyphilis are relatively rare, recognizing these uncommon subtypes of neurosyphilis and including them in one's syphilis illness script is crucial for primary care physicians and all clinicians caring for populations at risk for sexually transmitted infections. Finally, this case demonstrates that, in keeping with the protean nature of syphilis, neurosyphilis is a disease that can be particularly challenging to treat. Thus, consultation with an infectious disease expert is highly advisable in the workup and management of these patients.
